# Early diagnosis of intracranial atherosclerotic large vascular occlusion: A prediction model based on DIRECT-MT data

**DOI:** 10.3389/fneur.2022.1026815

**Published:** 2022-11-03

**Authors:** He Li, Hong-Yu Ma, Lei Zhang, Pei Liu, Yong-Xin Zhang, Xiao-Xi Zhang, Zi-Fu Li, Peng-Fei Xing, Yong-Wei Zhang, Qiang Li, Peng-Fei Yang, Jian-Min Liu

**Affiliations:** ^1^Emergency Room, Naval Hospital of Eastern Theater, Zhoushan, China; ^2^Neurovascular Center, Changhai Hospital, Naval Medical University, Shanghai, China

**Keywords:** intracranial atherosclerosis, prediction model, large vascular occlusion, etiology of ischemic stroke, DIRECT-MT

## Abstract

**Aims:**

This study aimed to build a prediction model to early diagnose intracranial atherosclerosis (ICAS)-related large vascular occlusion (LVO) in acute ischemic stroke patients before digital subtractive angiography.

**Methods:**

Patients enrolled in the DIRECT-MT trial (NCT03469206) were included in our secondary analysis and distributed into ICAS-LVO and non-ICAS-LVO groups. We also retrieved demographic data, medical histories, clinical characteristics, and pre-operative imaging data. Hypothesis testing was used to compare data of the two groups, and univariate logistic regression was used to identify the predictors of ICAS-LVO primarily. Then, we used multivariate logistic regression to determine the independent predictors and formulate the prediction model. Model efficacy was estimated by the area under the receiver operating characteristic (ROC) curve (AUC) and diagnostic parameters generated from internal and external validations.

**Results:**

The subgroup analysis included 45 cases in the ICAS-LVO group and 611 cases in the non-ICAS-LVO group. Variates with *p* < 0.1 in the comparative analysis were used as inputs in the univariate logistic regression. Next, variates with *p* < 0.1 in the univariate logistic regression were used as inputs in the multivariate logistic regression. The multivariate logistic regression indicated that the atrial fibrillation history, hypertension and smoking, occlusion located at the proximal M1 and M2, hyperdense artery sign, and clot burden score were related to the diagnosis of ICAS-LVO. Then, we constructed a prediction model based on multivariate logistics regression. The sensitivity and specificity of the model were 84.09 and 74.54% in internal validation and 73.11 and 71.53% in external validation.

**Conclusion:**

Our current prediction model based on clinical data of patients from the DIRECT-MT trial might be a promising tool for predicting ICAS-LVO.

## Introduction

Several large-scale randomized controlled trials have demonstrated that endovascular thrombectomy can effectively treat large vascular occlusion (LVO) (1). Intracranial atherosclerosis (ICAS)-related LVO (ICAS-LVO) is a major etiology of LVO, especially in Asian populations (2, 3).

Although endovascular thrombectomy has been proven safe and effective for ICAS-LVO, there are some differences between the endovascular treatment strategies for ICAS-LVO and other LVO etiologies (4). Refractory stenosis and instant re-occlusion after successful recanalization are much more frequent in ICAS-LVO, requiring more rescue therapies (4, 5). Furthermore, due to the particularity of the lesion, the first-line endovascular strategy for ICAS-LVO also differs from other etiologies (6–8). Therefore, an accurate and rapid diagnosis of ICAS-LVO is necessary to choose the appropriate endovascular strategy.

The most common strategy to verify the diagnosis of ICAS-LVO is based on the existence of remaining atherosclerotic stenosis on digital subtractive angiography (DSA) imaging during the procedure (9). However, the endovascular diagnosis of ICAS-LVO requires time, which might undermine the efficiency of the recanalization procedure. Hence, it is necessary to accurately distinguish ICAS-LVO from other ischemic stroke etiologies before groin puncture.

Previous studies have identified several predictors for ICAS-LVO based on retrospective studies (10). Medical histories, the severity of clinical presentations, laboratory examinations, and imaging based on computed tomography angiography (CTA) and magnetic resonance angiography (MRA) can be potential predictors for LVO etiologies. Herein, we identified independent predictors and constructed a prediction model for the pre-DSA diagnosis of ICAS-LVO based on DIRECT-MT data to achieve more reliable results than retrospective studies (11).

## Methods

### Data source of development dataset

We analyzed data from LVO patients enrolled in the DIRECT-MT trials, an investigator-initiated, multicenter, prospective, randomized, open-label trial (11). The first patient was included in February 2018 and the last was in July 2019. In the development dataset, we included all the patients in the DIRECT-MT trial. Briefly, patients who met the following criteria were included: (1) over 18; (2) NIHSSs ≥ 2; (3) eligible for both intravenous thrombolysis (IVT) and mechanical thrombectomy; (4) without intracranial hemorrhage; (5) with large vascular occlusion of the anterior circulation confirmed by CTA; (6) IVT could be administrated within 4.5 h after symptom onset; (7) signed informed consent. The exclusion criteria included: (1) suffering from pre-stroke disability; (2) any contra-indication for IVT. The ischemic stroke etiology was assessed based on the medical history, clinical features, and DSA results. The identification of ICAS-LVO was primarily based on DSA imaging, including (1) residual stenosis > 70% after first-line thrombectomy; (2) moderate residual stenosis with impairment of distal flow; (3) microcatheter “first-pass” effect during the procedure (2, 12). Then, patients were divided into two groups: ICAS-LVO and non-ICAS-LVO. The TRIPOD statement was followed while preparing this manuscript.

### Inclusion of clinical data

The following data of patients were included in detail: age, gender, medical histories of atrial fibrillation (AF), diabetes, mechanical aorta, and (or) mitral valve impairment, hypertension, hypercholesterolemia, previous ischemic stroke, peripheral artery diseases, and smoking. The CT or CTA imaging presentations included hyperdense artery signs, the existence of new hypodensity lesion, extracranial carotid artery stenosis, intracranial stenosis of other arteries, the location of intracranial artery occlusion, occlusion at other sites, anterior communicational artery (AcomA) development, Alberta Stroke Program Early CT Score (ASPECTS), clot burden scores (CBS), and collateral scores. The clinical presentations included baseline systolic blood pressure (SBP), Glasgow coma score (GCS), and National Institute of Health stroke scale (NIHSS) scores. The laboratory examinations comprised platelet counts, activated partial thromboplastin time (APTT), and international normalized ratio (INR). The total missing rate of each item was lower than 5%, and the missing data were filled by multiple imputations.

### Imaging data

All radiological imaging was assessed by an independent core lab blinded to the trial group assignments. Two independent readers evaluated all imaging, and a consensus reading was performed by a senior reader of each team in case of discrepancies.

### Data source of external validation dataset

Data for external validation were collected from the Changhai Neurovascular Center database. Consecutive patients admitted to our department from November 2013 to December 2018 who met the following criteria in this database were included in our study: (1) over 18; (2) lesion etiology was confirmed by DSA and recorded accurately; (3) diagnosis of ischemic stroke induced by LVO of anterior circulation; (4) the cause of the stroke was not dissection, moyamoya disease or vasculitis; (5) without missing necessary data.

### Statistical analysis

Statistical analyses were performed using SAS software v. 9.2 (SAS Institute). Python algorithms were used to formulate the final logistic regression model. Categorical variables are presented as counts and proportions, and continuous variables are presented as medians and interquartile ranges (IQRs). χ^2^ tests, adjusted χ^2^ tests, and Fisher's exact tests were used to compare categorical variables between two groups. Rank sum tests were conducted to compare continuous variables. In the univariate logistic regression analysis, variables with *p* < 0.1 were included in the comparative analysis. Variables with *p* < 0.1 in the univariate logistic regression analysis were used as input in the multivariate logistic regression analysis. The enter method was applied to conduct the primary multivariate logistic regression, and the stepwise method was used to optimize the predictive model. The Synthetic Minority Oversampling Technique (SMOTE) algorithm was used to adjust imbalanced data from DIRECT-MT. The predictive ability of the models was estimated by internal validation with the area under the receiver operating characteristic (ROC) curve (AUC) and diagnostic efficiency parameters (sensitivity, specificity, positive predictive value, and negative predictive value). These parameters were calculated again with the external validation data to evaluate the model further.

## Results

### Baseline characteristics

This study included 656 cases, 611 attributed to the non-ICAS-LVO group and 45 to the ICAS-LVO group. According to our demographic data, age [73 (61–77) vs. 63 (55–68), *p* < 0.0007] and proportion of male patients (55.3 vs. 71.1%, *p* = 0.0392) differed between the two groups (non-ICAS-LVO vs. ICAS-LVO). The medical history data indicated that the proportions of AF history (48.1 vs. 15.6%, *p* < 0.0001), hypertension history (58.8 vs. 77.8%, *p* = 0.0119), and smoking personal history (20.1 vs. 40.0%, *p* = 0.0017) also significantly differed. Additionally, the imaging data showed that the proportions of hyperdense artery sign (45.8 vs. 13.3%, *p* < 0.0001), location of artery occlusion (ICA: 37.3 vs. 9.1%; proximal M1: 25.3 vs. 63.6%; distal M1: 25.0 vs. 25.0%; M2: 12.4 vs. 2.3%, *p* < 0.0001), and CBS level [4 (2–5) vs. 5 (4–6), *p* = 0.0006] significantly differed between the two groups. Several laboratory examinations, such as platelet count [186 (156–226) vs. 208 (175–264), *p* = 0.0031] and international normalized ratio (INR) [1.03 (0.97–1.09) vs. 0.99 (0.95–1.06), *p* = 0.0288], also significantly differed between groups (Table 1).

**Table 1:** The comparison analysis of two groups.

	**ICAS-LVO**	**non-ICAS-LVO**	**Methods**	**Statistics**	***P*-value**
	**(*n =* 45)**	**(*n =* 611)**			
**Demographic**					
Age (Median, Q1–Q3)	63 (55–68)	73 (61–77)	Rank Sum Test	*Z =* 3.39	0.0007
Male (*n*, %)	32, 71.1%	338, 55.32%	Chi-square	4.25	0.0392
Female (*n*, %)	13, 28.9%	273, 44.7%	Chi-square		
**Medical Histores**					
Atrial fibrillation (*n*, %)	7, 15.6%	294, 48.1%	Chi-square	17.90	< 0.0001
Diabetes mellitus (*n*, %)	10, 22.2%	114, 18.7%	Chi-square	0.35	0.5556
Hypertension (*n*, %)	35, 77.8%	359, 58.8%	Chi-square	6.32	0.0119
Hypercholesterolemia (*n*, %)	0, 0	27, 4.4%	Adjusted Chi-square	1.11	0.2931
Myocardial infarction (*n*, %)	2, 4.4%	30, 4.9%	Adjusted Chi-square	0.00	1.0000
Previous ischemic stroke (*n*, %)	3, 6.7%	87, 14.2%	Chi-square	2.03	0.1542
History of peripheral artery disease (*n*, %)	0, 0	4, 0.65%	Fisher's exact test	–	1.0000
Mechanical aorta or mitral valve reparation (*n*, %)	1, 2.2%	12, 2.0%	Fisher's exact test	–	0.6065
Smoking (*n*, %)	18, 40.0%	123, 20.1%	Chi-square	9.81	0.0017
Anticoagulate drugs (*n*, %)	2, 4.4%	47, 7.69%	Adjusted Chi-square	0.26	0.6128
**CT or CTA imaging**					
Hyperdense artery sign (*n*, %)	6, 13.3%	280, 45.8%	Chi-square	18.00	< 0.0001
Exsistence of new hypodensity lesion (*n*, %)	31, 68.9%	398, 65.1%	Chi-square	0.26	0.6098
Extracranial cervical artery stenosis (–, %)	21, 46.7%	258, 42.2%	Chi-square	0.34	0.5609
Intracranial stenosis of other atery (*n*, %)	38, 84.4%	450, 73.7%	Chi-square	2.56	0.1093
Location of intracranial atery occlusion (*n*, %)			CMH test	34.23	< 0.0001
ICA	4, 9.1%	222, 37.3%			
Proximal M1	28, 63.6%	151, 25.3%			
Distal M1	11, 25.0%	149, 25.0%			
M2	1, 2.3%	74, 12.4%			
Occlusion at other sites (*n*, %)	0, 0	10, 1.6%	Fisher's exact test	–	1.0000
AcomA visble (*n*, %)	41, 91.1%	539, 88.2%	Chi-square	0.34	0.5581
ASPECTS (Median, Q1–Q3)	8.5 (7–10)	9 (7–10)	Rank Sum Test	*Z =* 0.48	0.6315
CBS (Median, Q1–Q3)	5 (4–6)	4 (2–5)	Rank Sum Test	*Z =* 3.41	0.0006
Collateral Score (Median, Q1–Q3)	1 (1–2)	1 (1–1)	Rank Sum Test	*Z =* 1.62	0.1054
**Clinical presentations**					
Baseline SBP (Median, Q1–Q3)	151 (132–166)	145 (131–162)	Rank Sum Test	*Z =* 1.09	0.2752
GCS (Median, Q1–Q3)	11 (10–15)	12 (9–14)	Rank Sum Test	*Z =* 0.49	0.6214
NIHSS (Median, Q1–Q3)	17 (12-20)	17 (13-22)	Rank Sum Test	*Z =* 1.04	0.2975
**Laboratory examinations**					
Platelets (Median, Q1–Q3)	208 (175–264)	186 (156–226)	Rank Sum Test	*Z =* 2.96	0.0031
APTT (Median, Q1–Q3)	30.75 (28.15–33.85)	30.10 (26.70–34.40)	Rank Sum Test	*Z =* 0.68	0.4973
INR (Median, Q1–Q3)	0.99 (0.95–1.06)	1.03 (0.97–1.09)	Rank Sum Test	*Z =* 2.19	0.0288

### Univariate logistic regression

The univariate logistic regression included variables with p < 0.1 in the comparison analysis. Being male [odds ratio (OR) = 1.982, 95% confidence interval (CI) = 1.020–3.851, *p* = 0.043], hypertension history (OR = 2.450, 95% CI = 1.191–5.038, *p* = 0.015), smoking personal history (OR = 2.650, 95% CI = 1.414–4.968, *p* = 0.002), occlusion located at proximal M1 segment (OR = 5.169, 95% CI = 2.722–9.816, *p* < 0.0001), and CBS levels (OR = 1.381, 95% CI = 1.155–1.652, *p* < 0.0001), and platelets count (OR = 1.005, 95% CI = 1.001–1.009, *p* = 0.01) were positively correlated to ICAS-LVO diagnoses. In contrast, AF history (OR = 0.199, 95% CI = 0.088–0.453, *p* < 0.0001), existence of hyperdense artery sign (OR = 0.182, 95% CI = 0.076–0.437, *p* < 0.0001), occlusion located at ICA (OR = 0.169, 95% CI = 0.060–0.478, *p* < 0.0001), and age (OR = 0.968; 95% CI = 0.946–0.990; *p* = 0.005) were negatively correlated to ICAS-LVO diagnoses (Table 2).

**Table 2:** Results of univariate logistic analysis.

	**Results**	**Value**	**95% CI**	***P*-value**
Age	OR	0.968	0.946, 0.990	0.005
Male	OR	1.982	1.020, 3.851	0.043
Atrial fibrillation	OR	0.199	0.088, 0.453	< 0.0001
Hypertension	OR	2.450	1.191, 5.038	0.015
Smoking	OR	2.650	1.414, 4.968	0.002
Hyperdense artery sign	OR	0.182	0.076, 0.437	< 0.0001
**Location of intracranial atery occlusion**				
ICA	OR	0.169	0.060, 0.478	0.001
Proximal M1	OR	5.169	2.722, 9.816	< 0.0001
Distal M1	OR	1.002	0.494, 2.032	0.995
M2	OR	0.164	0.022, 1.211	0.076
CBS	OR	1.381	1.155, 1.652	< 0.0001
Platelets	OR	1.005	1.001, 1.009	0.010
INR	OR	0.127	0.007, 2.243	0.159

### Multivariate logistic regression and model evaluation

Variables with *p* < 0.1 in the univariate logistic regression were further included in the multivariate logistic regression using the enter method. The primary logistic regression showed that AF (OR = 0.227, 95% CI = 0.088–0.585, *p* = 0.002), hypertension history (OR = 3.261, 95% CI = 1.390–7.647, *p* = 0.007), hyperdense artery sign (OR = 0.223, 95% CI = 0.084–0.589, *p* = 0.002), occlusion located at proximal M1 segment (OR = 5.323, 95% CI = 2.234–12.679, *p* < 0.0001), and M2 segment (OR = 0.074, 95% CI = 0.008–0.677, *p* = 0.021), and CBS (OR = 1.686, 95% CI = 1.243–2.285, *p* = 0.001) were independently correlated to ICAS-LVO diagnoses (Table 3).

**Table 3:** Results of multivariate logistic regression.

	**OR value**	**95% CI**	***P*-value**	**Stepwise**	**OR value**	**95% CI**	***P*-value**
				**adjustment**	**after adjustment**		
Age	0.987	0.960, 1.016	0.387	Exclude	–	–	–
Male	1.338	0.560, 3.197	0.512	Exclude	–	–	–
Atrial fibrillation	0.227	0.088, 0.585	0.002	Include	0.184	0.075, 0.451	< 0.0001
Hypertension	3.261	1.390, 7.647	0.007	Include	3.205	1.385, 7.416	0.006
Smoking	1.790	0.761, 4.210	0.182	Include	2.225	1.059, 4.676	0.035
Hyperdense artery sign	0.223	0.084, 0.589	0.002	Include	0.217	0.083, 0.567	0.002
**Location of intracranial atery occlusion**						
ICA	1.112	0.303, 4.084	0.872	Exclude	-	–	–
Proximal M1	5.323	2.234, 12.679	< 0.0001	Include	5.104	2.381, 10.941	< 0.0001
M2	0.074	0.008, 0.677	0.021	Include	0.073	0.009, 0.625	0.017
CBS	1.686	1.243, 2.285	0.001	Include	1.700	1.258, 2.300	0.001
Platelets	1.002	0.997, 1.007	0.466	Exclude	–	–	

The secondary logistic regression by stepwise method included AF history, hypertension history, personal smoking history, hyperdense artery sign, occlusion located at proximal M1 and M2 segments, and CBS in the final prediction model for ICAS-LVO (Table 3), as follows:


$$\begin{array}{l} y\;=-5.29427513-1.40472448x_{1}+0.98564739x_{2}\\ \;+\;0.74078982x_{3}+1.47468031x_{4}-1.498444x_{5}\\ \;-\;1.25189376x_{6}+0.45772141x_{7} \end{array}$$

where, *x*_1_*, x*_2_, *x*_3_, *x*_4_, *x*_5_, *x*_6_, and *x*_7_ represented AF history, hypertension history, personal smoking history, occlusion located at proximal M1, occlusion located at M2, hyperdense artery sign, and CBS, respectively. The dependent variable *y* was calculated by inputting the predictors *x*_1_ to *x*_7_ into the predictive model.

**Figure 1:**
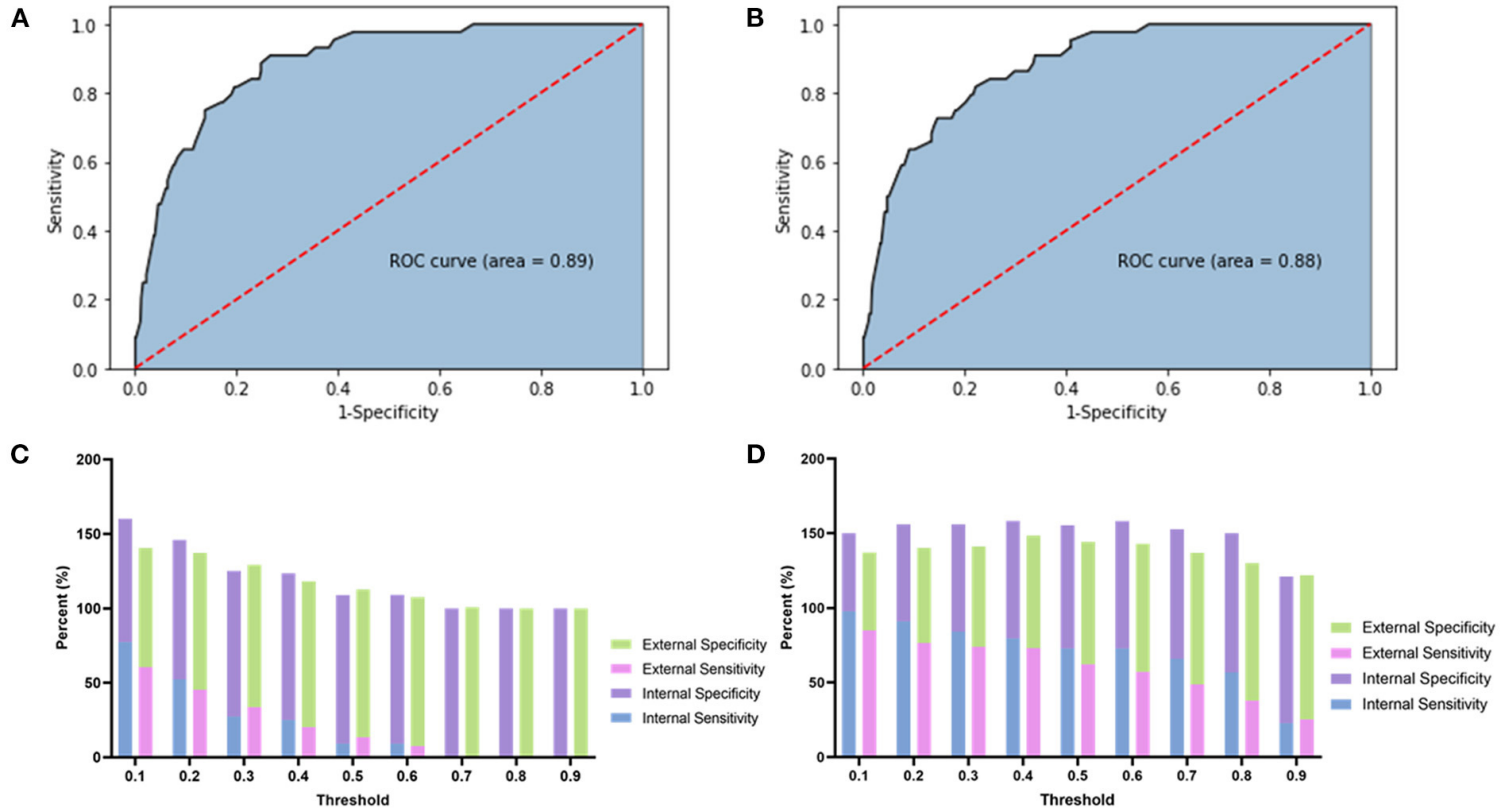
This figure showed the ROC, sensitivity, and specificity of each model. **(A)** The ROC of the original prediction model. **(B)** The ROC of the predictive regression model after SMOTE adjustment. **(C)** The stacked bar chart of the sensitivity and specificity of the original prediction model. **(D)** The stacked bar chart of the sensitivity and specificity of the adjusted prediction model.

The AUC of this model was 0.89 (Figure 1A). The model's sensitivity, specificity, positive predictive value, and negative predictive value at different thresholds are presented in Figure 1C and Supplementary Table 2. The optimal threshold value (0.069) was regarded as the one with which Youden's index of the model was maximum, and the evaluation parameters were 88.64%, 75.21%, 20.86%, and 98.90%, respectively.

Furthermore, the prediction model was tested by external validation data from the Neurovascular Center of Changhai Hospital (see Supplementary Table 1 for baseline characteristics). The sensitivity, specificity, positive predictive value, and negative predictive value for the prediction model were 68.91, 74.24, 51.90, and 85.55%, respectively (Supplementary Table 2).

### Multivariate logistic regression and model evaluation after SMOTE adjustment

To avoid the influence of imbalanced data on the final model, we used the SMOTE algorithm to adjust the logistic regression model as follows:


$$\begin{array}{l} y\;=-2.37879489-2.72026712x_{1}+0.66579282x_{2}\\ \;+\;0.22096829x_{3}+1.76203474x_{4}-3.59398673x_{5}\\ \;-\;2.42168058x_{6}+0.51568487x_{7} \end{array}$$

where, *x*_1_*, x*_2_, *x*_3_, *x*_4_, *x*_5_, *x*_6_, *and x*_7_ represented AF history, hypertension history, smoking personal history, occlusion located at proximal M1, occlusion located at M2, hyperdense artery sign, and CBS, respectively.

**Figure 2:**
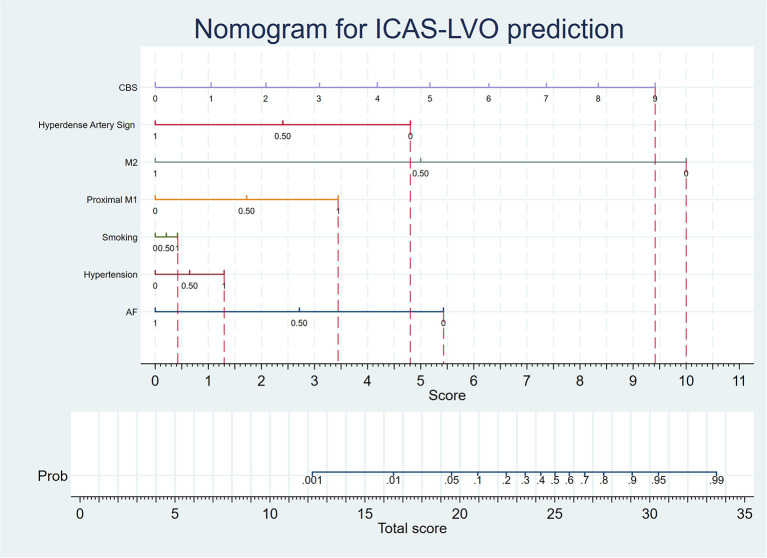
The nomogram is based on the adjusted prediction model. When predicting the etiology of LVO, the medical histories, imaging data, and CBS of the patient were collected. According to the nomogram, if the patient does not have an AF history, he will gain a 5.4 score. If the patient has a hypertension history, he will gain a 1.3 score. If the patient has a smoking personal history, he will gain a 0.4 score. If the patient has occlusion at the proximal M1 segment, he will gain a 3.4 score. If the patient does not have occlusion at the M2 segment, he will gain a 10 score. If the patient does not have a hypertense artery sign, he will gain a 4.8 score. The patient will gain 0, 1.1, 2.1, 3.1, 4.2, 5.2, 6.3, 7.3, 8.3, and 9.4 scores, if his CBS were 0, 1, 2, 3, 4, 5, 6, 7, 8, and 9 respectively. Afterward, the physician can calculate the total score of the patient, and the probability of ICAS-LVO diagnosis can be determined by the scale corresponding to the total score.

The AUC of this model was 0.88 (Figure 1B). The sensitivity, specificity, positive predictive value, and negative predictive value of the adjusted model at different thresholds are presented in Figure 1D and Supplementary Table 3. The optimal threshold of the adjusted model was 0.35. At this threshold, the evaluation parameters were 84.09, 74.54, 19.58, and 98.45%, respectively. In external validation, the evaluation parameters were 73.11, 71.53, 50.88, and 86.83% for the adjusted model. Finally, a nomogram was composed based on the final model to provide references to clinical practice (Figure 2).

## Discussion

Herein, we identified several factors independently correlated to ICAS-LVO diagnoses, including AF history, hypertension history, personal smoking history, occlusion located at proximal M1 and M2, hyperdense artery sign, and CBS. We constructed a predictive model for distinguishing ICAS-LVO from other etiologies based on multivariate logistic regression. After the SMOTE algorithm adjustment, the validation parameters of the adjusted prediction model were acceptable.

Several medical histories were correlated to ICAS-LVO diagnoses, and their diagnostic value has a physiological or pathological basis. For example, AF, the primary cause of cardioembolic (CE)-LVO, is a medical history negatively correlated to ICAS-LVO diagnoses (13). A patient suffering LVO with an AF history is suspected of having CE-LVO (14). However, this deduction is not always accurate in clinical practice. According to previous studies, the proportion of patients with AF history in the ICAS-LVO group ranged from 2.9 to 25.5% (10, 15, 16). Our preliminary data also indicated that more than 10% of ICAS-LVO patients suffered from AF. In the DIRECT-MT data, the proportion of patients with AF history in the ICAS-LVO group was 15.5% (7/45) and 10.9% (13/119) in the validation data. These data indicated that it was unwise to exclude the diagnosis of ICAS-LVO in patients with AF history. Although the AF history is a strong negative indicator of ICAS-LVO, more data is still required to provide a prediction model with higher accuracy.

Conversely, histories of hypertension and smoking were positively correlated to ICAS-LVO incidence. Hypertension and smoking histories have long been considered predictors of intracranial atherosclerosis (17). Additionally, hypertension enhances advanced atherosclerosis, decreases plaque stability, and induces cardiac death in hyperlipidemic rabbits (18). Smoking also contributes to the generation of oxidized low-density lipoprotein and the development of lipid metabolism impairment and atherosclerotic plaque and is positively correlated to symptomatic ICAS (19, 20). These data indicated that hypertension and smoking histories are predictors for ICAS-LVO and contribute to its development.

Imaging data based on CTA is also valuable to the early diagnosis of ICAS-LVO. CTA can indicate the location of occlusion before endovascular treatment. In clinical practice, the middle cerebral artery (MCA) is the most susceptive location to ICAS. According to the SAMMPRIS trial, the proportion of MCA atherosclerosis was 43.7%, higher than the proportion of internal carotid artery (ICA), vertebral artery (VA), and basilar artery (BA) atherosclerosis (21). Studies have also indicated that the proportion of proximal M1 occlusion was higher in ICAS-LVO patients, and the ratio of distal M1 occlusion was higher in CE-LVO patients (15, 16). These data supported our finding that the occlusion at the proximal M1 segment was an independent predictor for ICAS-LVO. In contrast, the occlusion located at the proximal M2 segment negatively predicted ICAS-LVO. According to Lee et al. the proportion of the occlusion located at the M2 segment of MCA was 3.0% in ICAS-LVO patients and 10.9% in embolic LVO patients (16). Jia et al. also showed that patients with ICAS-LVO had a lower M2 segment occlusion ratio than those with embolic LVO (15). These results indicated that the M2 segment of MCA is not the predictive site of ICAS-LVO, which can predict LVO etiology. The CBS based on CTA is another predictor for ICAS-LVO and is consistent with its characteristics. It had been reported that the clot burden was lower in an occlusion induced by stenotic intracranial artery than by cardioembolism (12). Besides, long-term artery stenosis might generate better collateral circulation in ICAS-LVO patients, reducing the CBS (22).

Hyperdense artery sign is an imaging manifestation observed on CT plain scanning and is negatively correlated to ICAS-LVO incidence. This parameter was identified in the 1990s and was initially used to predict poor outcomes or complications after ischemic stroke (23). Afterward, the hyperdense artery sign was also related to a higher red blood cell content in the clot (24). Berge et al. and Leys et al. indicated that the hyperdense artery sign is always present in embolic stroke patients (23, 25). Kuo et al. showed that large-artery atherosclerotic occlusion patients had a lower positive hyperdense artery sign rate than those with cardioembolic occlusion (26). Thus, we hypothesized that the hyperdense artery sign resulted from the accumulation of more red blood cells caused by a higher clot burden, which deserves further investigation.

In the present study, we built a prediction model with independent ICAS-LVO predictors. The model reached the maximum Youden's index with a sensitivity of 88.64% and specificity of 75.21% at the threshold of 0.069 in the internal validation. However, the sensitivity and specificity of this model were only 68.91 and 74.24% in the external validation, far from clinical practice requirements. This phenomenon might be related to the overfitting of the current model (27). The overfitting might be due to the lack of subjects in the DIRECT-MT trial and the imbalanced grouping derived from the lower ICAS-LVO incidence. We could not solve the first problem limited to the study design, while the second one was resolvable by balancing the data. Thus, we applied the SMOTE algorithm to balance the subject numbers of the ICAS-LVO and non-ICAS-LVO groups (28). The final model showed a maximum Youden's index with a sensitivity of 84.09% and a specificity of 74.54% in the internal validation, slightly lower than the original model. The sensitivity and specificity at the new model threshold were improved to 73.11 and 71.53%, and the highest were 73.11 and 75.25%. The distribution of the sensitivity and specificity at different thresholds was also more reasonable than the original model, which could help clinical practice.

However, our current study also has some limitations. Although we built the first model based on clinical trial data to distinguish ICAS-LVO from other etiologies, the sensitivity and specificity of the model remained relatively low. The first reason was that the number of subjects included in the DIRECT-MT study was insufficient to build a prediction model with higher efficacy. Secondly, parameters with predictive value to ICAS-LVO were not included in this study, such as C-reactive protein serum levels and the radiological morphology of the occlusion site. In the future, more subjects and parameters should be included to build a more effective and reliable prediction model of ICAS-LVO.

## Conclusion

In summary, we identified several independent predictors of ICAS-LVO, including AF history, hypertension history, smoking personal history, occlusion located at proximal M1 and M2, hyperdense artery sign, and CBS. An effective prediction model based on these predictors was built, and the final AUC, sensitivity, and specificity of the model were 0.88, 73.11, and 71.53%, respectively. This model provided a powerful tool to differentiate ICAS-LVO from other LVO etiologies. Nevertheless, further study is still required to improve the efficacy of the prediction model.

## Data availability statement

The raw data supporting the conclusions of this article will be made available by the authors, without undue reservation.

## Ethics statement

The studies involving human participants were reviewed and approved by the all relevant Local Ethics Committees and research boards of DIRECT-MT trial (NCT03469206). The list is available as a Supplementary File. All the participants assigned the consents. The patients/participants provided their written informed consent to participate in this study.

## Author contributions

HL composed the manuscript. H-YM collected the clinical data and modified the manuscript. LZ performed the analysis with Python. PL performed the analysis with Stata 15. Y-XZ composed Tables 1–3. X-XZ composed Supplementary Tables. Y-WZ composed Figure 1. P-FY and QL raised the idea and provided directions to HL and PL. J-ML composed Figure 2. All authors contributed to the article and approved the submitted version.

## Funding

The DIRECT-MT trial was funded by the Stroke Prevention Project of the National Health Commission of the People's Republic of China and the Wu Jieping Medical Foundation. This subgroup analysis was funded by the Natural Science Foundation of China (No. 82071278), Distinguished and Outstanding Yong Scholar Cultivation Project of Changhai Hospital (No. 2021JCSQ03), and Young Medical Talent Cultivation and Funding Project of Shanghai (No. 20224Z0008).

## Conflict of interest

The authors declare that the research was conducted in the absence of any commercial or financial relationships that could be construed as a potential conflict of interest.

## Publisher's note

All claims expressed in this article are solely those of the authors and do not necessarily represent those of their affiliated organizations, or those of the publisher, the editors and the reviewers. Any product that may be evaluated in this article, or claim that may be made by its manufacturer, is not guaranteed or endorsed by the publisher.
