# Supplementary material for: Early diagnosis of intracranial atherosclerotic large vascular occlusion: A prediction model based on DIRECT-MT data
Source: Front Neurol. 2022 Nov 3;13:1026815. doi: 10.3389/fneur.2022.1026815 (PMC9670732; doi:10.3389/fneur.2022.1026815)
Supplement: Supplementary file 1 [file Data_Sheet_1.docx]

**Supplementary Table 1**

The baseline characteristics of the validation data.

|  | ICAS-LVO (n=119) | non-ICAS-LVO (n=295) | Methods | Statistics | p-value |
| --- | --- | --- | --- | --- | --- |
| Demographic |  |  |  |  |  |
| Age (Median, Q1-Q3) | 66 (59-74) | 71 (63-78) | Rank Sum Test | Z=3.032 | 0.0024 |
| Male (n, %) | 95, 79.8% | 151, 51.2% | Chi-square | 28.86 | <0.0001 |
| Female (n, %) | 24, 20.2% | 144, 48.8% | Chi-square |  |  |
| Medical Histories |  |  |  |  |  |
| Atrial fibrillation (n, %) | 13, 10.9% | 193, 65.4% | Chi-square | 100.74 | <0.0001 |
| Diabetes Mellitus (n, %) | 30, 25.2% | 35, 11.9% | Chi-square | 11.41 | 0.0007 |
| Hypertension (n, %) | 87, 73.1% | 155, 52.5% | Chi-square | 14.77 | <0.0001 |
| Hypercholesterolemia (n, %) | 6, 5.0% | 4, 1.3% | Adjusted Chi-square | 4.89 | 0.0270 |
| Coronary Diseases (n, %) | 10, 8.4% | 56, 19.0% | Adjusted Chi-square | 7.08 | 0.0078 |
| Previous Ischemic Stroke (n, %) | 17, 14.3% | 30, 10.2% | Chi-square | 1.43 | 0.2322 |
| Smoking (n, %) | 56, 47.1% | 42, 14.2% | Chi-square | 50.56 | <0.0001 |
| Drinking (n, %) | 4, 3.4% | 10, 3.4% | Adjusted Chi-square | 0.00 | 0.9884 |
| CT or CTA imaging |  |  |  |  |  |
| Hyperdense Artery Sign (n, %) | 24, 20.2% | 115, 39.0% | Chi-square | 13.46 | 0.0002 |
| Location of Intracranial Artery Occlusion (n, %) |  |  |  |  |  |
| ICA | 42, 35.3% | 117, 39.7% | Chi-square | 0.68 | 0.4084 |
| Proximal M1 | 58, 48.7% | 112, 37.8% | Chi-square | 4.07 | 0.0437 |
| Distal M1 | 3, 2.5% | 22, 7.5% | Chi-square | 3.64 | 0.0563 |
| M2 | 16, 13.5% | 44, 14.9% | Chi-square | 0.15 | 0.7006 |
| ASPECTS (Median, Q1-Q3) | 9 (8-10) | 9 (7-10) | Rank Sum Test | Z=-2.955 | 0.0031 |
| CBS (Median, Q1-Q3) | 5 (4-6) | 4 (2-5) | Rank Sum Test | Z=-5.256 | <0.0001 |
| Clinical Presentations |  |  |  |  |  |
| GCS (Median, Q1-Q3) | 13 (10-15) | 11 (8-14) | Rank Sum Test | Z=-4.209 | <0.0001 |
| NIHSS (Median, Q1-Q3) | 13 (7-18) | 18 (13-22) | Rank Sum Test | Z=6.478 | <0.0001 |
| Laboratory examinations |  |  |  |  |  |
| Platelets (Median, Q1-Q3) | 211 (174-265) | 192 (160-231) | Rank Sum Test | Z=-3.519 | 0.0004 |

**Supplementary Table 2**

The sensitivity, specificity, positive predictive value, and negative predictive value of the prediction model at different thresholds.

|  | Internal Validation | | | | | External Validation | | | | |
| --- | --- | --- | --- | --- | --- | --- | --- | --- | --- | --- |
|  | Sensitivity | Specificity | Positive Predictive Value | Negative Predictive Value | Youden's Index | Sensitivity | Specificity | Positive Predictive Value | Negative Predictive Value | Youden's Index |
| Threshold=0.069* | 88.64% | 75.21% | 20.86% | 98.90% | 63.85% | 68.91% | 74.24% | 51.90% | 85.55% | 43.14% |
| Threshold=0.1 | 77.27% | 82.91% | 25.00% | 98.02% | 60.19% | 60.50% | 80.00% | 54.96% | 83.39% | 40.50% |
| Threshold=0.2 | 52.27% | 93.63% | 37.70% | 96.38% | 45.91% | 45.38% | 91.86% | 69.23% | 80.65% | 37.24% |
| Threshold=0.3 | 27.27% | 97.82% | 48.00% | 94.81% | 25.10% | 33.61% | 95.59% | 75.47% | 78.12% | 29.21% |
| Threshold=0.4 | 25.00% | 98.49% | 55.00% | 94.69% | 23.49% | 20.17% | 97.97% | 80.00% | 75.26% | 18.13% |
| Threshold=0.5 | 9.09% | 99.83% | 80.00% | 93.71% | 8.92% | 13.45% | 99.32% | 88.89% | 73.99% | 12.77% |
| Threshold=0.6 | 9.09% | 100.00% | 100.00% | 93.72% | 9.09% | 7.56% | 100.00% | 100.00% | 72.84% | 7.56% |
| Threshold=0.7 | 0.00% | 100.00% | 0.00% | 93.14% | 0.00% | 0.84% | 100.00% | 100.00% | 71.43% | 0.84% |
| Threshold=0.8 | 0.00% | 100.00% | 0.00% | 93.14% | 0.00% | 0.00% | 100.00% | 0.00% | 71.26% | 0.00% |
| Threshold=0.9 | 0.00% | 100.00% | 0.00% | 93.14% | 0.00% | 0.00% | 100.00% | 0.00% | 71.26% | 0.00% |

**Supplementary Table 3**

The sensitivity, specificity, positive predictive value, and negative predictive value of the adjusted prediction model at different thresholds.

|  | Internal Validation | | | | | External Validation | | | | |
| --- | --- | --- | --- | --- | --- | --- | --- | --- | --- | --- |
|  | Sensitivity | Specificity | Positive Predictive Value | Negative Predictive Value | Youden's Index | Sensitivity | Specificity | Positive Predictive Value | Negative Predictive Value | Youden's Index |
| Threshold=0.1 | 97.73% | 52.26% | 13.11% | 99.68% | 49.99% | 84.87% | 52.20% | 41.74% | 89.53% | 37.08% |
| Threshold=0.2 | 90.91% | 64.99% | 16.06% | 98.98% | 55.90% | 76.47% | 63.73% | 45.96% | 87.04% | 40.20% |
| Threshold=0.3 | 84.09% | 71.86% | 18.05% | 98.39% | 55.95% | 73.95% | 67.12% | 47.57% | 86.46% | 41.07% |
| Threshold=0.35* | 84.09% | 74.54% | 19.58% | 98.45% | 58.63% | 73.11% | 71.53% | 50.88% | 86.83% | 44.63% |
| Threshold=0.4 | 79.55% | 78.73% | 21.60% | 98.12% | 58.27% | 73.11% | 75.25% | 54.37% | 87.40% | 48.36% |
| Threshold=0.5 | 72.73% | 82.58% | 23.53% | 97.62% | 55.31% | 62.18% | 82.03% | 58.27% | 84.32% | 44.22% |
| Threshold=0.6 | 72.73% | 85.43% | 26.89% | 97.70% | 58.15% | 57.14% | 85.76% | 61.82% | 83.22% | 42.91% |
| Threshold=0.7 | 65.91% | 86.77% | 26.85% | 97.19% | 52.68% | 48.74% | 88.14% | 62.37% | 81.00% | 36.88% |
| Threshold=0.8 | 56.82% | 93.13% | 37.88% | 96.70% | 49.95% | 37.82% | 92.20% | 66.18% | 78.61% | 30.02% |
| Threshold=0.9 | 22.73% | 98.32% | 50.00% | 94.52% | 21.05% | 25.21% | 96.61% | 75.00% | 76.20% | 21.82% |
