# Supplementary material for: Early diagnosis of intracranial atherosclerotic large vascular occlusion: A prediction model based on DIRECT-MT data
Source: Front Neurol. 2022 Nov 3;13:1026815. doi: 10.3389/fneur.2022.1026815 (PMC9670732; doi:10.3389/fneur.2022.1026815)
Supplement: Supplementary file 2 [file Data_Sheet_2.docx]

List of Ethical Committees

Ethical committee of Changhai hospital, Naval Medical University

Ethical committee of Affiliated Hospital of Nantong University

Ethical committee of The Second Affiliated Hospital of Zhejiang University

Ethical committee of The First People's Hospital of Hangzhou

Ethical committee of TaiZhou First People's Hospital

Ethical committee of Zhangzhou Affiliated Hospital of Fujian Medical University

Ethical committee of The First Affiliated Hospital of Jinan University

Ethical committee of Henan Provincial People’s Hospital

Ethical committee of Luoyang Central Hospital Affiliated to Zhengzhou University

Ethical committee of The First Hospital of Hebei Medical University

Ethical committee of Linyi People’s Hospital

Ethical committee of Yantaishan Hospital

Ethical committee of Liaocheng People’s Hospital

Ethical committee of The First Affiliated Hospital of Jilin University

Ethical committee of The First People’s Hospital of Changzhou

Ethical committee of Nanjing Drum Tower Hospital

Ethical committee of Nanjing First Hospital

Ethical committee of The First Affiliated Hospital of Soochow University

Ethical committee of Jiangsu Province Hospital

Ethical committee of Daping Hospital

Ethical committee of Chongqing Three Gorges Central Hospital

Ethical committee of Nanning Second People’s Hospital

Ethical committee of Zhoukou Central Hospital

Ethical committee of The First Affiliated Hospital of Harbin Medical University

Ethical committee of Central Hospital of Baotou

Ethical committee of Anhui Provincial Hospital

Ethical committee of Sichuan Provincial Hospital

Ethical committee of Tongji Hospital

Ethical committee of Ningbo First Hospital

Ethical committee of Wenzhou Central Hospital

Ethical committee of Hunan Provincial People's Hospital

Ethical committee of Binhai Hospital

Ethical committee of Qingdao Center Hospital

Ethical committee of First Affiliated Hospital of Anhui Medical University

Ethical committee of The Affiliated Hospital of Guizhou Medical University

Ethical committee of Maoming People’s Hospital

Ethical committee of Nanyang City Center Hospital

Ethical committee of Zhuhai People’s Hospital

Ethical committee of The Second Affiliated Hospital of Guangzhou Medical University

Ethical committee of The Second Affiliated Hospital of Bengbu Medical University

Ethical committee of Zhengzhou Central Hospital
